# Supplementary figures and images for: Microbiome Research: Open Communication Today, Microbiome Applications in the Future
Source: Microorganisms. 2020 Dec 10;8(12):1960. doi: 10.3390/microorganisms8121960 (PMC7763060; doi:10.3390/microorganisms8121960)

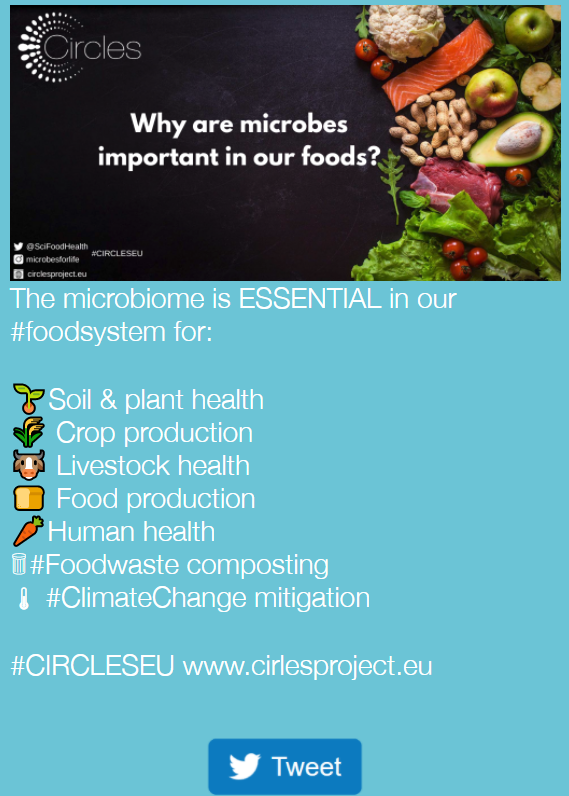

Supplement: Supplementary file 1 [file microorganisms-08-01960-s001.zip › Supplementary Material/S1a.png]

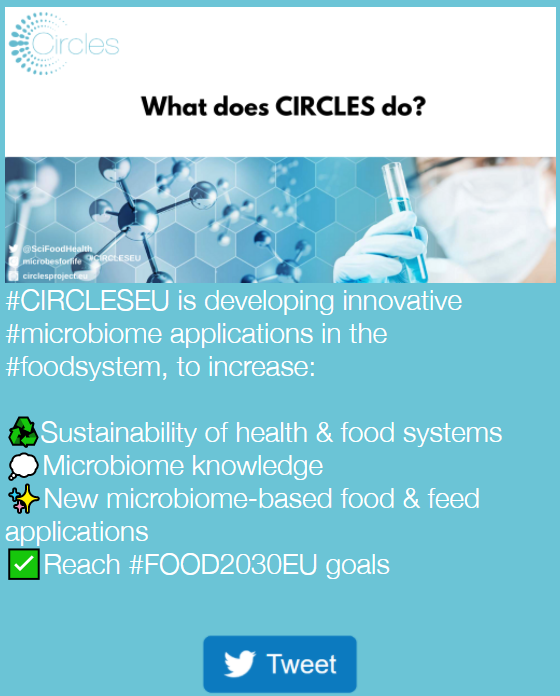

Supplement: Supplementary file 1 [file microorganisms-08-01960-s001.zip › Supplementary Material/S1b.png]

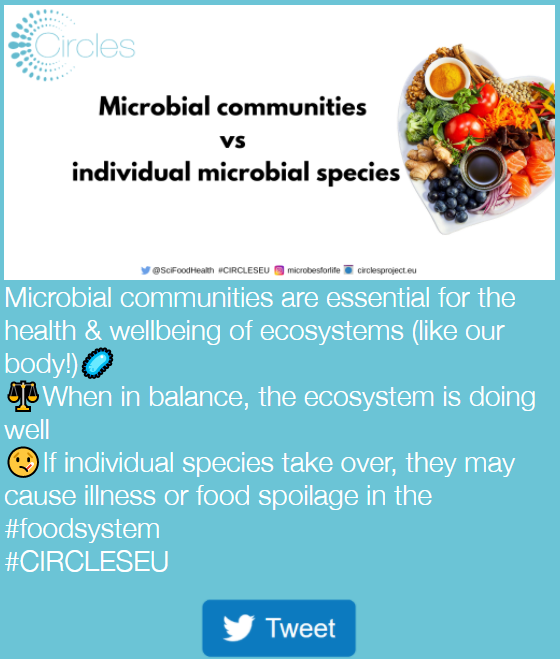

Supplement: Supplementary file 1 [file microorganisms-08-01960-s001.zip › Supplementary Material/S1c.png]
